# KRDS: a web server for evaluating drug resistance mutations in kinases by molecular docking

**DOI:** 10.1186/s13321-018-0274-y

**Published:** 2018-04-10

**Authors:** Aeri Lee, Seungpyo Hong, Dongsup Kim

**Affiliations:** 1 Department of Bio and Brain Engineering, KAIST, Daejeon, 34141 Republic of Korea; 2 Division of Nutrition and Metabolism Research, Korea Food Research Institute, Wanju-gun, Jeollabuk-do Republic of Korea

**Keywords:** Drug resistance, Kinase, Docking, Ensemble, Point mutation

## Abstract

Kinases are major targets of anti-cancer therapies owing to their importance in signaling processes that regulate cell growth and proliferation. However, drug resistance has emerged as a major obstacle to cancer therapy. Resistance to drugs has various underlying mechanisms, including the acquisition of mutations at drug binding sites and the resulting reduction in drug binding affinity. Therefore, the identification of mutations that are relevant to drug resistance may be useful to overcome this issue. We hypothesized that these mutations can be identified by combining recent advances in computational methods for protein structure modeling and ligand docking simulation. Hence, we developed a web-based tool named the Kinase Resistance Docking System (KRDS) that enables the assessment of the effects of mutations on kinase-ligand interactions. KRDS receives a list of mutations in kinases, generates structural models of the mutants, performs docking simulations, and reports the results to users. The changes in docking scores and docking conformations can be analyzed to infer the effects of mutations on drug binding and drug resistance. We expect our tool to improve our understanding of drug binding mechanisms and facilitate the development of effective new drugs to overcome resistance related to kinases; it may be particularly useful for biomedical researchers who are not familiar with computational environments. Our tool is available at http://bcbl.kaist.ac.kr/KRDS/.

**Electronic supplementary material:**

The online version of this article (10.1186/s13321-018-0274-y) contains supplementary material, which is available to authorized users.

## Background

Kinases constitute approximately 30% of human cellular proteins and are involved in the transmission of cellular signals by transferring a phosphate group to their specific targets [1, 2]. Specifically, kinases play important roles in the regulation of cell growth and proliferation and therefore they are potential targets for anti-cancer therapies; kinase inhibitors have been successfully developed into anti-cancer drugs [3–5]. However, cancer cells often acquire resistance to these drugs after prolonged treatment, and the prevention of drug resistance has become a major challenge in anti-cancer therapy development [6, 7]. Drug resistance is the result of diverse mechanisms, including an imbalance between drug influx and efflux, alterations in drug targets, and the activation of alternative pathways [8]. However, the alteration of drug binding sites is a direct and well-known mechanism of drug resistance [7–9] and missense mutations at binding sites in kinases, such as ABL1, EGFR, FLT3, KIT and PDGFRA, have been observed in various cancers [7, 10]. For example, a threonine (T) to isoleucine (I) substitution at residue 315 (T315I) of BCR-ABL1 results in reduced sensitivity to a number of drugs, including imatinib, nilotinib, dasatinib, and bosutinib, in patients with chronic myeloid leukemia [11, 12]. The T790M mutation in the epidermal growth factor receptor (EGFR) kinase domain is also responsible for the resistance of non-small cell lung cancer cells to erlotinib and gefitinib [13, 14]. A better understanding of the structural mechanism of drug resistance will aid in the development of new drugs. For example, ponatinib was designed to treat patients who exhibited resistance to previously available drugs, such as imatinib, nilotinib, dasatinib, and bosutinib [15]. Therefore, the identification of mutations responsible for drug resistance would not only reveal the mechanism of drug resistance, but also lead to the development of new effective drugs.

Efforts to understand drug resistance have led to the compilation of various types of information about drug resistance in databases [16–21]. Sandgren et al. [16] established a well-curated public database containing mutations related to tuberculosis drug resistance, and users can find information on how often certain mutations are observed for particular drugs (http://www.tbdreamdb.com). The Comprehensive Antibiotic Research Database (CARD) (http://arpcard.mcmaster.ca) integrates the sequence data for microbial antibiotic resistance genes [17]. CARD assigns putative antibiotic resistance genes to unannotated microbial genome sequences based on sequence similarity. The Stanford HIV Drug Resistance Database (HIVDB) (http://www.bioafrica.net/saturn) contains reverse transcriptase sequences underlying HIV drug resistance [18]. Sibley and Ringwald [19] outlined how to construct a publicly accessible database containing information related to antimalarial drug resistance. In the Cancer Drug Resistance Database (CancerDR), the pharmacological profiles of 148 anticancer drugs against around 1000 cancer cell lines were deposited with 116 drug-target relations [20]. The association between various mutations and Herceptin in H342-positive breast cancer can be found in the HerceptinR database [21]. These databases offer a list of mutations and drug-target associations. Researchers can determine if a certain mutation is associated with drug resistance based on the information in these databases, and the structural changes caused by mutations can be integrated.

None of the resistance databases mentioned above provide an automated prediction tool to study structural changes leading to drug resistance caused by mutations in human kinases. Our server searches for mutations related to drug resistance by assessing mutational effects on drug binding. This can be achieved by modeling the structure of mutants computationally and by performing molecular docking simulations. Alcaro et al. [22] evaluated significantly correlated resistance mutations in HIV reverse transcriptase (RT) with three non-nucleoside RT inhibitors (NNRTIs) using AutoDock Vina. Sivaprakasam et al. [23] examined double mutations (A16V + S108T) that occurred in dihydrofolate reductase (DHFR) of PT (PfDHFR-TS) and led to resistance to specific drugs. They used four docking programs (FlexX, GOLD, Glide, and Molegro) to investigate the performance of docking and the correlation of docking scores based on the binding affinity data between wild-type and mutant-type forms. They obtained the best correlation (*R*^2^ = 0.911) between docking scores and binding affinity data using the GOLD program.

**Fig. 1:**
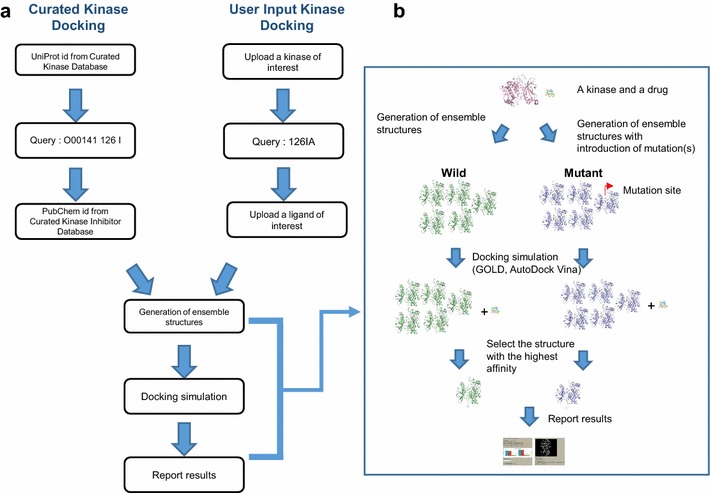
Server workflow. **a** Users can submit a list of mutations and a list of drugs via the Curated Kinase Docking and User Input Kinase Docking sections. The Curated Kinase Docking accesses the list of mutations in the kinases and the list of ligands in our Database, for which we have collected data of kinases with a known structure. Users can upload the structures of kinases and ligands through the User Input Kinase Docking section. **b** Following submission, the server will model the mutant structures and perform docking simulations. An example of a simple schematic diagram for predicting drug resistance with one kinase and one drug using our server is shown. In the original kinase structure, the wild-type generates five ensemble structures, while the mutant type introduces mutations and then generates five ensemble structures. After that, our server performs a molecular docking simulation using GOLD and AutoDock Vina. When the simulation is finished, the docking scores with the highest affinity and the corresponding conformations of the original and mutant kinases are reported to users

Currently, there are a number of protein modeling and docking tools that can be combined to evaluate the structural impact of mutations on drug binding, but for many biomedical researchers, using these tools would be a major challenge. Therefore, we developed a web server named the Kinase Resistance Docking System (KRDS) that allows researchers to easily evaluate the effect of a mutation on drug binding. Our server automatically generates conformational ensembles for both wild-type and mutant-type forms using RosettaBackrub [24], and performs docking simulations of given drugs to both types using GOLD [25] and AutoDock Vina [26]. The docking scores and conformations of the original and mutant kinases are reported to users (Fig. 1). We expect our server to be used as a tool to obtain structural models for studies of drug resistance.

## Implementation

### Data

The structures of human protein kinases were downloaded from the RCSB Protein Data Bank (PDB) (http://www.rcsb.org/pdb/) [27] based on the Swiss-Prot human kinase list released in 2016 (http://www.uniprot.org/docs/pkinfam). The list of UniProtKB/Swiss-Prot human protein kinases was divided into families or subgroups according to the sequence similarity of catalytic domains. We chose the structure with the best resolution if there were multiple structures. Water molecules, unnecessary heteroatoms, and solvent and solute molecules from all PDB files of 241 kinases were removed, and structural alignments were generated. We next integrated additional structural information on the 241 kinases, such as DFG-in and DFG-out conformations [28]. Users can browse detailed information on the 241 kinases, e.g., gene symbol Entrez IDs, PDB IDs, resolution, mutation status, and in or out states of DFG, in our Curated Kinase Database. A list of 178 commercially available kinase inhibitors used by Anastassiadis et al. [29] for comprehensive kinase activity profiles was used to establish a kinase inhibitor database. Among the 178 kinase inhibitors, a quinazoline scaffold (erlotinib, gefitinib, and lapatinib), a thiazole scaffold (dasatinib), aminopyrimidine scaffold (crizotinib), and a pyrrolo[2,3-*d*]pyrimidine scaffold (tofacitinib) as Type I inhibitors, an indolone scaffold (sunitinib) as a type I $${\raise0.7ex\hbox{$1$} \!\mathord{\left/ {\vphantom {1 2}}\right.\kern-0pt} \!\lower0.7ex\hbox{$2$}}$$ B inhibitor, and a 2-phenylaminopyrimidine scaffold (imatinib) as a Type II inhibitor were observed [30, 31]. The structures of kinase inhibitors were retrieved from PubChem (http://pubchem.ncbi.nlm.nih.gov/) [32]. In total, the structures of 241 kinases and 178 kinase inhibitors were deposited in our server.

### Structural ensemble generation and mutant structure modeling

In conventional molecular docking simulations, the structures of ligands are treated as flexible, while the structures of proteins are treated as rigid [33, 34]. The lack of flexibility in protein structures can hinder the search for the proper binding conformation. One way to solve this problem is to use an ensemble of protein structures during docking simulations [35]. Thus, in our tool, an ensemble of five structures is generated using a flexible backbone modeling method, RosettaBackrub [24, 36], for both the original and the mutant kinase structures. Only five conformational ensembles for each structure are generated to minimize computational time (30–40 min for one drug). After the dockings are performed on those five conformations, the best docked structure is chosen. The mutations are incorporated into the models by substituting the amino acids during structure modeling. We generated five non-native CDK2 structures with the RosettaBackrub algorithm and performed re-docking of co-crystals into the corresponding five ensembles (Additional file 1: Table S1). Variation in RMSD values between ensemble structures, such as 2FVD, 4ERW, and 4GCJ, was observed. The results showed that the best docked ensemble had the lowest RMSD poses, except for 3TI1 in complex with sunitinib. When we compared our docking results with kinase binding assay data [37], the docking result for staurosporine were consistent with experimental data, while the binding result for CDK2 with sunitinib was not determined in a 10 micromole screen, and dinaciclib, r547, and x64 were not available in assay data. The high RMSD values between CDK2 and sunitinib for the re-docking of sunitinib to the tertiary CDK2 ensembles might be explained by the weak interaction, as shown in the experimental result.

### Molecular docking

GOLD version 5.2.2 [25] and AutoDock Vina version 1.1.2 [26] display good docking performance according to previous studies [38–40] and therefore were adopted for molecular docking. The genetic algorithm and GoldScore fitness function are employed in GOLD. The GoldScore scoring function is based on terms from molecular mechanics force fields which calculates the sum of the interaction terms. For AutoDock Vina, the default conformation search algorithm, which is a combination of optimization algorithms, including genetic algorithms, particle swarm optimization, and simulated annealing, and the default scoring function, which is a hybrid score function derived from the X-score, are used. The hybrid scoring function in AutoDock Vina is a combination of empirical and knowledge-based functions. The empirical scoring function considers energy terms, such as hydrogen bonds, ionic interactions, hydrophobic effects, and binding entropy. Each energy component is used to generate a final score. The knowledge-based scoring function is based on a statistical analysis of the frequency distributions of favorable interactions between a ligand and a protein observed from crystal structures. A series of docking simulations are performed for each structure in the ensembles, and the binding pose with the highest docking score is reported.

### Results report

**Fig. 2:**
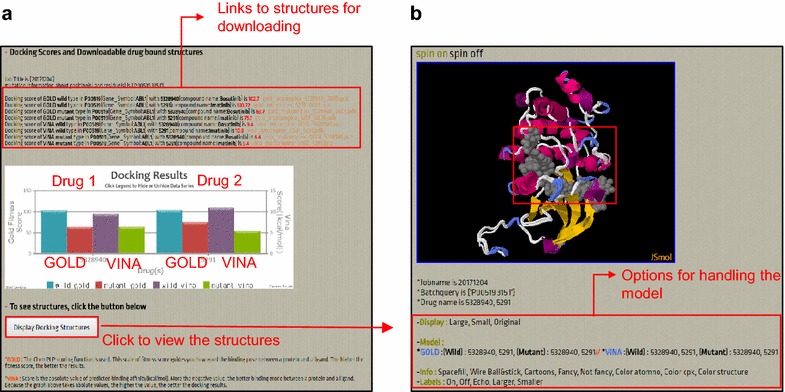
Result report. **a** Docking scores for original and mutant kinases. The above example shows the results of docking with two drugs in Curated Kinase Docking. **b** The docking conformations for original and mutant kinases are illustrated using JSmol

The results of the docking simulation are reported to the user via email. First, the docking scores with both the original and mutant kinases are reported for the quantitative evaluation of the effects of mutations on ligand binding (Fig. 2a). GOLD uses force-field-based scoring functions [25], while AutoDock Vina uses its own hybrid scoring function [26]. GOLD outputs docking results in terms of a fitness score. The higher the fitness is, the better the docked interaction between a protein and a ligand. AutoDock Vina outputs a result in terms of docking energy scores. The lower the score is, the better the docked interaction. Our server reports the fitness score of GOLD and the absolute values of AutoDock Vina. If the two docking systems do not agree in terms of docking scores, running our program multiple times and examining the binding poses of a ligand would be recommended. The docking conformations of original and mutant kinases are visualized using JSmol (http://wiki.jmol.org/index.php/JSmol) to highlight the structural consequence of the mutation (Fig. 2b). Users can download these conformations for further structural comparisons.

### Method selection

**Fig. 3:**
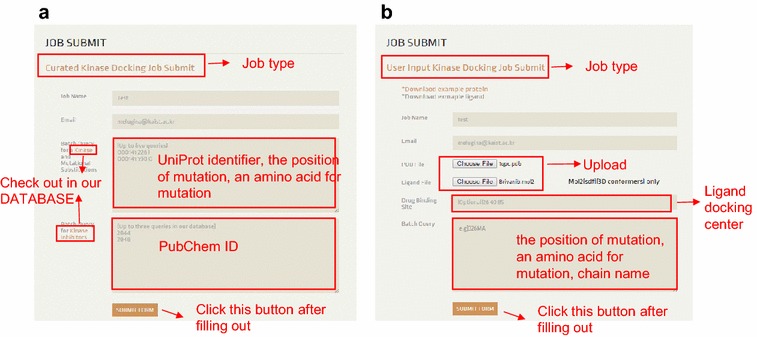
Job submission page. **a** In Curated Kinase Docking, the UniProt ID, residue position, and mutant amino acid should be specified in Kinase and Mutational Substitutions. The PubChem ID is allowed in Batch Query for Kinase Inhibitors. **b** In User Input Kinase Docking, users can upload a PDB format file for a kinase structure and a MOL2 or SDF format file for a ligand structure, which should be of a three-dimensional conformer. Specification of the drug binding site will result in docking around the given three-dimensional coordinate. The list of mutations in the form of chain name, residue index, and mutation amino acid should be given in the Mutation List text box

Users can select one of three options in our server: Curated Kinase Docking, User Input Kinase Docking, and Database. Users can use our automated mutant structure modeling and ligand docking simulation services in the first two options (Fig. 3). In the Database option, users can browse the list of human kinases and kinase inhibitors.

### Curated kinase docking

**Fig. 4:**
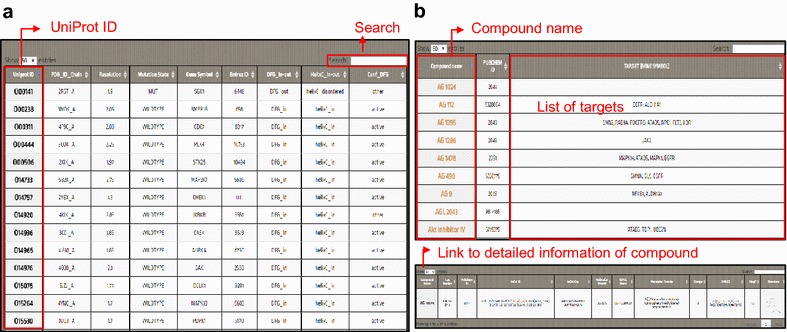
Kinase and kinase inhibitor database. **a** The Curated Kinase Database contains 241 human protein kinases and the UniProt identifier, mutation state of PDB, gene symbol, DFG-in or DFG-out state, etc. **b** The Kinase Inhibitor Database provides information on 178 kinase inhibitors. When the user clicks on the compound name, detailed information, such as molecular weight, SMILES, InChl identifier, and structures of the drug, will be provided

In this option, users can model and perform docking simulations for mutations in the kinases in the curated Database. Users need to enter the following information: (1) Job name, (2) email address, (3) list of mutations, and (4) list of ligands (Fig. 4a). Upon submission, the server will generate a structural model for each mutation and perform docking simulations between all of the listed ligands and kinase mutation structures, including the original kinase structure. The results will be reported to the user through the given email address with the given job name. The list of mutations has a specific format, in which a mutation should be described on a line with a UniProt identifier to specify a kinase, the position of mutation, and the amino acid to be mutated. For example, “O00141 226 I” represents a mutation in kinase SGK1 that substitutes residue 226 with isoleucine. For the ligand list, the PubChem identifier of the ligand should be given. For a single submission, up to five mutations and up to two drugs can be given. In this option, both the kinases and drugs have to be in our database, which can be found in the DATABASE section. Otherwise, users can upload the structure of a kinase and a drug through User Input Kinase Docking.

### User Input Kinase Docking

Users can manually upload the structures of a kinase and a drug in this option, and designate mutation sites in the kinase. Users can search specialized information about kinases at http://www.kinase.com, which provides classified human kinase genes that are not in our datasets, and those kinases can be used to model kinases in this section. In this section, users need to upload the kinase and drug structure files and enter the following information: (1) Job name, (2) email address, (3) list of mutations, and (4) ligand docking coordinates (Fig. 4b). A mutation should be input as a single string of the name of the chain, position of the mutation, and amino acid to be mutated. For example, “366MA” indicates the mutation of residue 366 on chain A into methionine. Additionally, users can designate the coordinates of the docking center, on which the docking simulation will be performed. Otherwise, the uploaded kinase will be superimposed with the reference kinase (PDB ID: 4BKJ), and the ligands will be docked around its ligand binding site. The kinase input file should be in PDB format, and the ligand file should be in Mol2 or SDF (3D conformer) format. Docking simulation for up to five mutations is allowed, and the Curated Kinase Docking procedure will be executed.

### Database

We have collected the structures of human kinases and the structures of kinase inhibitors from recent publications. This database contains 241 human kinases and offers gene information, including the UniProt identifier, mutation state, gene symbol, Entrez identifier, and structural information on kinases, such as the PDB identifier and DFG-in or DFG-out state (Fig. 4a). This database also contains 178 kinase inhibitors that were deposited in our system along with their name, PubChem identifier, and drug targets (Fig. 4b). Users can search kinases and drugs in the database before executing docking mutations in Curated Kinase Docking.

## Case studies

### Re-docking simulations

Before applying our method, we first confirmed that the molecular docking on kinases mostly reproduced the correct binding poses by re-docking ligands into the original co-crystal positions, including the kinases that we used for the case study (Additional file 1: Table S2 and Figure S1).

### Structural docking simulations of DFG states

We then checked whether the molecular docking approach reflected the various conformations of kinases and yielded reliable docking results. It is well known that protein kinases adopt two “Asp-Phe-Gly (DFG)” motifs: the active DFG-in conformation and inactive DFG-out conformation at the activation loop [41]. Kinase inhibitors are classified depending on which state of the kinase conformation the drug binds to [30, 41]. We wanted to determine if the consideration of DFG-in and DFG-out states for kinases would be reflected in molecular docking. We docked on various conformations of five kinases based on well-organized DFG-in and DFG-out state data [28]. Additional file 1: Table S3 lists the various kinase structures used for docking for these five kinases. The docking values shown in Additional file 1: Table S4 are the average docking scores over all available PDB structures in each state of the kinase. For example, imatinib is a Type II kinase inhibitor that binds to the kinase when the DFG motif is in the “out” state [41]. The docking results of imatinib showed higher values for structures of the DFG-out state than of the DFG-in state. Likewise, we observed that molecular docking generated moderate docking scores in general for the various conformations of kinases in each state (Additional file 1: Table S4, Figure S2).

We next examined whether these results are reproduced by our system in which the docking calculations were performed by taking a single PDB structure from DFG-in and from DFG-out, generating ensembles, conducting dockings on ensembles with ligands, and extracting maximum values among ensembles for each ligand. As shown in Additional file 1: Table S5, our docking system generally reproduced the docking results when we utilized all PDB structures (Additional file 1: Table S4).

### Mutant docking simulations

**Fig. 5:**
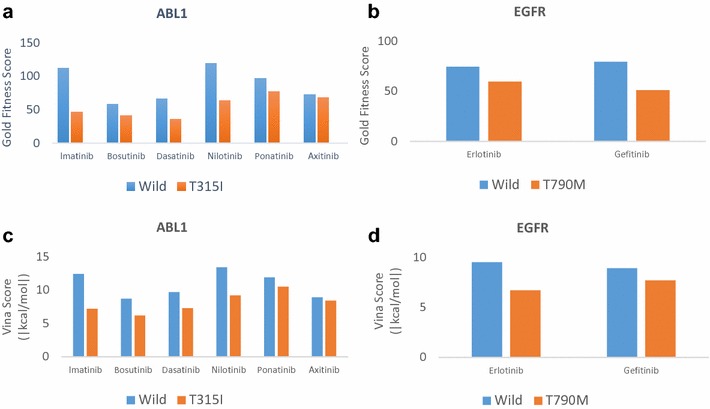
BCR-ABL1-T315I and EGFR-T790M docking scores. **a** The Gold fitness scores of the T315I-sensitive drugs imatinib, dasatinib, nilotinib, and bosutinib decreased by more than 20% compared to those of the wild type. However, the docking scores of the T315I-insensitive drugs ponatinib and axitinib remained higher than those of other T315I-sensitive drugs. **b** The EGFR-T790M mutant is known to be responsible for resistance to erlotinib and gefitinib, and their docking scores decreased by 20 and 36%, respectively, in the docking simulation. **c** The absolute values of the Vina scores (kcal/mol) of T315I decreased by 41.93, 28.73, 24.74, and 31.24% for imatinib, bosutinib, dasatinib, and nilotinib, respectively, compared to those of the wild type. The docking scores of T315I for ponatinib and axitinib decreased by 11.76 and 5.61%, respectively. However, the docking score of ponatinib for T315I was already high, with an absolute value of 10.5 kcal/mol. **d** The absolute values of Vina scores (kcal/mol) of T790M decreased by 29.13 and 13.48% for erlotinib and gefitinib, respectively, compared to those of the wild type

**Fig. 6:**
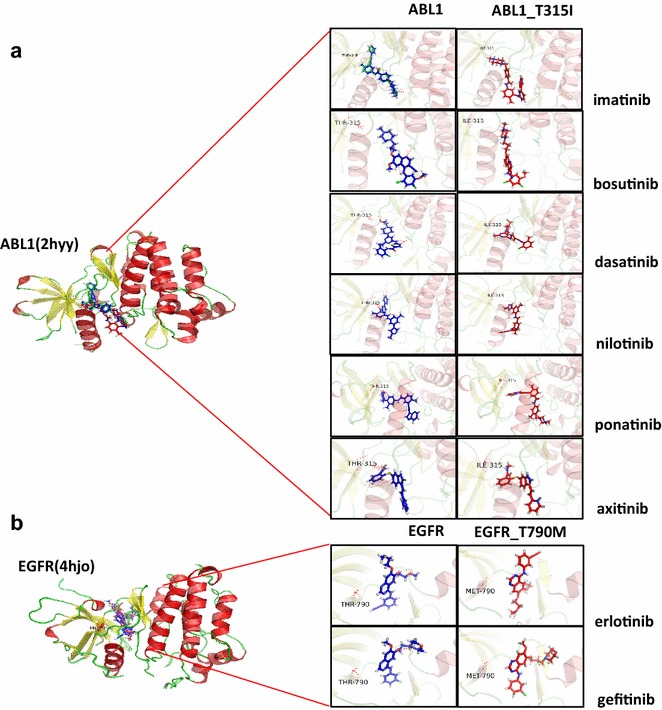
Predicted binding mode from the docking analysis. **a** The predicted binding conformations of each drug for both wild-type and mutant ABL1. **b** The predicted binding conformations of each drug for both the wild-type and mutant EGFR

Our server provides a collection of services that generate the structural ensembles of the original and mutant kinases and perform docking simulations. This approach will enable the assessment of the effects of mutations on drug binding by analyzing changes in docking scores and in docking conformations induced by a certain mutation. To show the validity of this approach, we applied our method to two well-known mutations responsible for drug resistance. The T315I mutation in BCR-ABL1 was reported to be responsible for the resistance of chronic myeloid leukemia to several anti-cancer drugs [7]. This mutation has emerged following treatment with imatinib, and therefore mutant cells are resistant to imatinib [15]. In addition, it has resulted in resistance to other anti-cancer drugs, including dasatinib, nilotinib, and bosutinib. However, the mutant is still susceptible to ponatinib and axitinib [42]. We applied our method to predict this drug-dependent response of the T315I mutation. We used the human ABL1 kinase domain (Uniprot ID: P00519) in complex with imatinib (PDB ID: 2HYY). According to the docking simulation in GOLD, the T315I mutation reduced the binding affinities of imatinib, dasatinib, nilotinib, and bosutinib by 59, 46, 47, and 30%, respectively (Fig. 5a, Additional file 1: Table S6). On the other hand, the docking scores for the susceptible drugs, ponatinib and axitinib, remained higher, although binding affinity was reduced by 20 and 5% for ponatinib and axitinib, respectively. The results from Vina in ABL1 were consistent with those from GOLD (Fig. 5c, Additional file 1: Table S7). The absolute values of the Vina score (kcal/mol) for T315I decreased from 12.4 to 7.2 for imatinib (41.93% reduction), from 8.7 to 6.2 for bosutinib (28.73% reduction), from 9.7 to 7.3 for dasatinib (24.74% reduction), and from 13.4 to 9.2 for nilotinib (31.34% reduction). The Vina results for ponatinib and axitinib decreased from 11.9 to 10.5 (11.76% reduction) and from 8.9 to 8.4 (5.61% reduction), respectively. The predicted binding conformations of imatinib, bosutinib, and dasatinib appeared to change significantly after the threonine residue was substituted with isoleucine, suggesting that the threonine at position 315 plays an important role in stabilizing drug binding in ABL1 (Fig. 6a). Ponatinib was unaffected by the mutation of the threonine residue and interacts with other residues. The pose of axitinib in mutant ABL1 was not significantly different from that of the wild type, because axitinib is capable of interacting with isoleucine.

The T790M mutation of EGFR is another example of a drug resistance mutation, and it was reported to be responsible for the resistance of non-small lung cancer to gefitinib and erlotinib [7]. We used the human EGFR kinase domain (Uniprot ID: P00533) in complex with erlotinib (PDB ID: 4HJO). The docking scores in GOLD for both drugs were decreased by the T790M mutation from 74.46 to 59.78 for gefitinib (20% reduction) and from 79.53 to 51.15 for erlotinib (36% reduction) (Fig. 5b, Additional file 1: Table S6). The results from Vina for EGFR were also consistent with those from GOLD (Fig. 5d, Additional file 1: Table S7). The absolute values of the Vina score (kcal/mol) for T790M decreased from 9.5 to 6.7 for erlotinib (29.47% reduction) and from 8.9 to 7.7 for gefitinib (13.48% reduction). The predicted binding conformation of erlotinib flipped back after the substitution of the threonine at position 790 with a methionine residue (Fig. 6b). The threonine residue at position 790 appears to be involved in forming a stable bond with a ligand. According to our docking analyses, the drug resistance-related mutations could be identified from the changes in the predicted binding affinity. When taken together, we observed a difference of more than 20% in binding affinity when we analyzed two well-known kinases with eight drugs (Additional file 1: Table S9) that are related to drug resistance.

### Docking simulations for comparison with experimental values

**Fig. 7:**
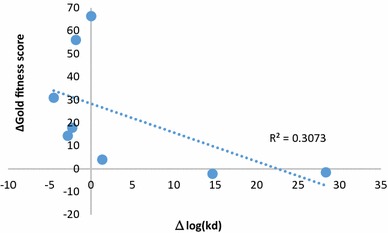
Correlation between docking scores and experimental results for ABL1(T315I), EGFR(T790M), and RET(V804M). The *x*-axis represents the log value of $k_{d}$ obtained by subtracting the mutant-type log $k_{d}$ values from the wild-type $k_{d}$ log values. The *y*-axis shows the Gold fitness scores obtained by subtracting the mutant-type docking scores from the wild-type docking scores

To further validate our system, we conducted a comparative analysis of our docking scores with kinase binding assay data [37]. There are nine other kinases with mutation information from kinase binding assay data, i.e., BRAF, FGFR3, FLT3, KIT, MET, PIK3CA, RET, MET, and LRRK2. A crystal structure is not available for LRRK2. No drugs showed a significant $k_{d}$ value between the wild-type and mutant BRAF, FGFR3, FLT3, MET, and PIK3CA. Excluding these kinases, dockings of ABL1, EGFR, KIT, and RET were performed for each kinase based on mutation information within our system, and the docking scores were compared with the $k_{d}$ values (Additional file 1: Table S8). If the $k_{d}$ value of a drug is higher for the mutant than for the wild-type kinase, the docking result should be lower for the mutant than for the wild type. As expected, a negative correlation was detected between changes in docking score and $k_{d}$ value following mutations (Fig. 7), but the correlation was not statistically significant (*R*^2^ =0.3073; *p* value = 0.45). Additional $k_{d}$ values for kinases associated with drug resistance are necessary to confirm this correlation.

## Conclusions

Following treatment with anti-cancer drugs, cancer cells gradually acquire mutations that negate the beneficial effects of the drugs. The growth of these cancer cells can no longer be inhibited, and drug resistance becomes a major threat to the survival of patients. The identification of the mutations responsible for drug resistance is the first step in resolving this problem. In this study, we present a computational analysis of structural modeling of both wild-type and mutant kinases with kinase inhibitors based on molecular docking simulations and provide a publicly accessible web server. This server would be particularity useful for biomedical researchers who are not familiar with the computational environment. We anticipate that researchers will utilize our tool to explore the predicted binding mode of kinase inhibitors with structurally modeled mutant kinases.

## Additional file

**Additional file 1: Table S1.** Re-docking of five ligands co-crystalized with CDK2 to the five RosettaBackrub generated CDK2 conformations. **Table S2**. RMSD values after re-docking of co-crystals into native structures. **Table S3**. The list of pdb ids of DFG-in and its corresponding DFG-out structures to perform docking in ABL1, BRAF, EGFR, FGFR4, and IGF1R. **Table S4**. The averaged docking values of DFG-in and its corresponding DFG-out structures. **Table S5**. The maximum docking values obtained among ensembles. **Table S6**. The docking results of ABL1 and EGFR using GOLD. **Table S7**. The docking results of ABL1 and EGFR using AutoDock Vina. **Table S8**. Comparison of docking scores and kinase activity data in ABL1 and EGFR. **Table S9**. Tanimoto coefficient scores between two drugs. **Figure S1**. Re-docking of imatinib and erlotinib in ABL1 and EGFR. **Figure S2**. The results of re-docking ligands on different DFG states.
