# Supplementary material for: KRDS: a web server for evaluating drug resistance mutations in kinases by molecular docking
Source: J Cheminform. 2018 Apr 10;10:20. doi: 10.1186/s13321-018-0274-y (PMC5891443; doi:10.1186/s13321-018-0274-y)
Supplement: Supplementary file 1 — Additional file 1: Table S1. Re-docking of five ligands co-crystalized with CDK2 to the five RosettaBackrub generated CDK2 conformations. Table S2. RMSD values after re-docking of co-crystals into native structures. Table S3. The list of pdb ids of DFG-in and its corresponding DFG-out structures to perform docking in ABL1, BRAF, EGFR, FGFR4, and IGF1R. Table S4. The averaged docking values of DFG-in and its corresponding DFG-out structures. Table S5. The maximum docking values obtained among ensembles. Table S6. The docking results of ABL1 and EGFR using GOLD. Table S7. The docking results of ABL1 and EGFR using AutoDock Vina. Table S8. Comparison of docking scores and kinase activity data in ABL1 and EGFR. Table S9. Tanimoto coefficient scores between two drugs. Figure S1. Re-docking of imatinib and erlotinib in ABL1 and EGFR. Figure S2. The results of re-docking ligands on different DFG states. [file 13321_2018_274_MOESM1_ESM.docx]

**Supplementary Table S1.** Re-docking of five ligands co-crystalized with CDK2 to the five RosettaBackrub generated CDK2 conformations.

| **Drugs**  **(PDB ID)** | **Dinaciclib**  **(4KD1)** | **R547**  **(2FVD)** | **Staurosporine**  **(4ERW)** | **Sunitinib**  **(3TI1)** | **X64**  **(4GCJ)** |
| --- | --- | --- | --- | --- | --- |
| **Affinity** | NA | Kd: 0.5nM | Kd: 7nM | Kd: > 10uM**^*^** | IC50: 20nM |
| **Conformation 1**† | 93.19  (0.755)‡ | 53.88  (9.432) | 69.39  (5.744) | 60.59  (9.216) | **70.4**  **(0.949)** |
| **Conformation 2**† | **97.99**  **(0.634)** | 56.16  (9.878) | 82.97  (1.198) | **69.64**  **(10.697)^*^** | 62.26  (2.802) |
| **Conformation 3**† | 94.92  (0.995) | **63.45**  **(1.052)** | 74.82  (1.165) | 61.59  (10.533) | 60.5  (9.414) |
| **Conformation 4**† | 88.85  (0.755) | 52.95  (9.669) | 75.7  (1.356) | 68.19  (10.533) | 61.2  (3.122) |
| **Conformation 5**† | 95.08  (0.528) | 59.63  (2.886) | **83.59**  **(1.371)** | 57.15  (11.141) | 57.05  (8.982) |
| **†** The protein conformations were generated by using RosettaBackrub algorithm with CDK2 structure with PDB IDs(4KD1, 2FVD, 4ERW, 3TI1, and 4GCJ) as the structural templates.  **‡** The GoldScore fitness function was used. Values in parentheses indicate RMSD values.  ***** The docking with sunitinib was failed. However, it binds to CDK2 only at very high concentration (Kd > 10uM) indicating its mode of binding is distinctively different from other ligands, probably because it was found by an attempt to find a ligand showing allosteric effect ([1](#_ENREF_1)). | | | | | |

**Supplementary Table S2**. RMSD values after re-docking of co-crystals into native structures.

| **Kinase** | **PDB** | **DFG state** | **RMSd†** | **Drug** |
| --- | --- | --- | --- | --- |
| **AAK1** | 4WSQ | **In** | **0.628** | **KSA** |
| **ABL1** | 2HYY | **Out** | **0.983** | **STI** |
| **AKT1** | 3OCB | **In** | **1.003** | **XM1** |
| **BRAF** | 3PRI | **In** | **0.879** | **0T2** |
| **BUB1** | 4QPM | **In** | **0.963** | **ADP** |
| **CAMK1** | 4FG8 | **In** | **1.129** | **ATP** |
| **CDK8** | 4F6U | **Out** | **0.576** | **0SR** |
| **CHEK1** | 4FSR | **In** | **0.472** | **HKC** |
| **CSK** | 1BYG | **Out** | **1.421** | **STU** |
| **CSNK1D** | 4KB8 | **In** | **0.648** | **1QN** |
| **CSNK1E** | 4HNI | **Out** | **0.993** | **16W** |
| **CSNK1G3** | 4G17 | **In** | **0.84** | **0VN** |
| **DAPK1** | 4TXC | **In** | **1.08** | **38G** |
| **DAPK3** | 3BQR | **In** | **1.295** | **4RB** |
| **DDR1** | 4BKJ | **Out** | **0.593** | **STI** |
| **DYRK1A** | 4MQ2 | **In** | **0.45** | **2C4** |
| **EGFR** | 4HJO | **In** | **1.252** | **AQ4** |
| **FES** | 3CD3 | **In** | **1.064** | **STU** |
| **FGFR1** | 5AM6 | **NA** | **0.722** | **38O** |
| **FGFR4** | 4UXQ | **Out** | **0.39** | **0LI** |
| **GAK** | 4C57 | **In** | **0.885** | **FEF** |
| **GSK3B** | 4ACD | **In** | **1.689** | **GR9** |
| **ITK** | 3MJ2 | **In** | **1.174** | **MJG** |
| **KDR** | 3VHK | **Out** | **1.112** | **BPK** |
| **KIT** | 3G0E | **Out** | **1.014** | **B49** |
| **NEK7** | 2WQN | **Out** | **1.618** | **ADP** |
| **NTRK2** | 4AT5 | **Out** | **1.299** | **MUJ** |
| **PAK1** | 4EQC | **In** | **0.882** | **XR1** |
| **PAK4** | 4O0Y | **In** | **0.366** | **2OO** |
| **PRKCA** | 3IW4 | **In** | **1.952** | **LW4** |
| **PRKCH** | 3TXO | **In** | **0.878** | **07U** |
| **PTK2B** | 4H1M | **Out** | **0.866** | **0YJ** |
| **TBK1** | 4EUU | **In** | **1.477** | **BX7** |
| **TEK** | 3L8P | **In** | **0.627** | **0CE** |
| **TYK2** | 4PY1 | **NA** | **1.443** | **2YK** |
| †The GoldScore fitness function was used. | | | | |

**Supplementary Table S3.** The list of pdb ids of DFG-in and its corresponding DFG-out structures to perform docking in ABL1, BRAF, EGFR, FGFR4 and IGF1R

| Kinase | DFG-in | DFG-out |
| --- | --- | --- |
| ABL1 | 2F4J,2GQG,2HZ4,2V7A,2G1T,  2G2I | 1IEP,1OPL,2E2B,2FO0,2G2H,  2HIW,2HYY,2HZ0,2HZI,3CS9,  3PYY,3QRI,3QRJ,3QRK,3UE4,  2G2F |
| BRAF | 4CQE,4WO5,4E26,2FB8,3C4C,  3D4Q,3OG7,3PPJ,3PPK,3PRF,  3PRI,3PSB,3PSD,3Q4C,3SKC,  3TV4,3TV6,4E4X,4EHE,4EHG,  4FK3,4H58,4MBJ,4MNF,4PP7,  4MNE | 1UWH,1UWJ,3IDP,3II5,3Q96,  4DBN,4FC0,4G9C,4G9R,4JVG,  4KSP,4KSQ |
| EGFR | 4RIY,4RIX,4RIW,4RJ4,4RJ5,  4RJ6,4RJ7,4RJ8,1M14,1M17,  1XKK,2EB2,2EB3,2GS2,2GS7,  2ITN,2ITO,2ITP,2ITQ,2ITT,  2ITU,2ITV,2ITW,2ITX,2ITY,  2ITZ,2J5E,2J5F,2J6M,2JIT,  2JIU,2JIV,2RGP,3BEL,3GT8,  3IKA,3LZB,3POZ,3UG1,3UG2,  3VJN,3VJO,3W2O,3W2P,3W2Q,  3W2R,3W2S,3W32,3W33,4G5J,  4G5P,4HJO,4I22,4I23,4I24,  4JQ7,4JQ8,4JR3,4JRV,4LI5,  4LL0,4LQM,4LRM,4R5S,4TKS,  4WKQ,4WRG,2GS6,2RFD,2RFE | 3GOP,4I1Z,4I20,2RF9,4I21 |
| FGFR4 | 4QQJ,4QQT,4TYE,4TYG,4TYI | 4UXQ,4QQ5,4QQC,4QRC,4R6V,  4TYJ |
| IGF1R | 2ZM3,3D94,3F5P,3LVP,3QQU,  1K3A | 1JQH,1M7N,1P4O,2OJ9,3I81,  3LW0,3NW5,3NW6,3NW7,3O23 |
|  | | |

**Supplementary Table S4.** Docking results of DFG-in and its corresponding DFG-out structures. The docking scores are the averaged values from various structures in each state.

|  | Vina Score (\|kcal/mol\|) | | GOLD Fitness Score | |
| --- | --- | --- | --- | --- |
| **ABL1** | **DFG-in†** | **DFG-out†** | **DFG-in** | **DFG-out** |
| Imatinib | 8.72 | **10.44** | 66.65 | **85.8** |
| VX-680 | 8.1 | 8.91 | 65.07 | 73.63 |
| **BRAF** | **DFG-in** | **DFG-out** | **DFG-in** | **DFG-out** |
| GDC0879 | **9.26** | 8.46 | **74.66** | 72.08 |
| Sorafenib | 9.08 | **10.92** | 61.31 | **77.06** |
| T1Q | 8.84 | 8.95 | 78.78 | 79.6 |
| **EGFR** | **DFG-in** | **DFG-out** | **DFG-in** | **DFG-out** |
| Erlotinib | 6.85 | 6.92 | **65.25** | 34 |
| Gefitinib**‡** | 7.58 | 6.82 | 65.72 | 31.17 |
| Lapatinib | **8.76** | 6.9 | **83.11** | 39.91 |
| Afatinib | **7.79** | 6.34 | **59.94** | 41.89 |
| Dacomitinib | **8.23** | 5.82 | **62.93** | 42.37 |
| WZ4002 | **8.27** | 5.72 | **64.09** | 44.94 |
| **FGFR4** | **DFG-in** | **DFG-out** | **DFG-in** | **DFG-out** |
| Dovitinib | 7.7 | 8.45 | 44.59 | 65.22 |
| Ponatinib | 6.82 | **10.55** | 23.36 | **87.03** |
| **IGF1R** | **DFG-in** | **DFG-out** | **DFG-in** | **DFG-out** |
| CCX | 8.8 | 8.15 | 68.39 | 66.55 |
| D94 | **9.65** | 9.16 | **68.3** | 60.84 |
| † The yellow shaded area represents the DFG-in structures and their associated ligands. The green shaded area represents the DFG-out structures and their associated ligands.  ‡ Shaded in half means that the ligand is observed in structures of both states. | | | | |

**Supplementary Table S5**.Docking results generated in our system. The docking scores are the maximum values extracted among ensembles.

|  | Vina Score (\|kcal/mol\|) | | GOLD Fitness Score | |
| --- | --- | --- | --- | --- |
| **ABL1** | 2F4J | 1IEP | 2F4J | 1IEP |
| Imatinib | 9 | **12.9** | 70.37 | 111.92 |
| VX-680 | **9.7** | 9.3 | **87.68** | 76.52 |
| **BRAF** | 2FB8 | 4KSP | 2FB8 | 4KSP |
| GDC0879 | **9.8** | 8.3 | **82.54** | 67.47 |
| Sorafenib | 8.7 | **10.4** | 60.19 | **78.20** |
| T1Q | 7.3 | 9.4 | **71.82** | 66.92 |
| **EGFR** | 3POZ | 3GOP | 3POZ | 3GOP |
| Erlotinib | **7.7** | 6.6 | **72.54** | 51.82 |
| Gefitinib | 8.7 | 6.7 | 73.45 | 68.01 |
| Lapatinib | **10.1** | 8.1 | **102.01** | 67.26 |
| Afatinib | **9.5** | 6.5 | **76.06** | 58.49 |
| Dacomitinib | **9.8** | 7.3 | **72.36** | 57.49 |
| WZ4002 | **9.0** | 7.8 | **73.44** | 50.34 |
| **FGFR4** | 4QQT | 4QRC | 4QQT | 4QRC |
| Dovitinib | **8.6** | 7.5 | 57.57 | 62.79 |
| Ponatinib | 8.4 | **10.8** | 60.97 | **87.12** |
| **IGF1R** | 1K3A | 1P4O | 1K3A | 1P4O |
| CCX | 8.7 | 4.5 | 63.38 | **65.10** |
| D94 | 8.2 | 8.63 | 56.68 | 59.21 |

**Supplementary Table S6**. The docking results of ABL1 and EGFR using Gold. The unit of the scores is fitness score.

| **ABL1** | **Wild** | **T315I** |
| --- | --- | --- |
| Imatinib | 112.65 | 46.35 |
| Bosutinib | 58.65 | 40.84 |
| Dasatinib | 66.76 | 35.92 |
| Nilotinib | 119.38 | 63.39 |
| Ponatinib | 96.65 | 77.36 |
| Axitinib | 72.49 | 68.58 |
| **EGFR** | **Wild** | **T790M** |
| Erlotinib | 74.46 | 59.78 |
| Gefitinib | 79.53 | 51.15 |

**Supplementary Table S7.** The docking results of ABL1 and EGFR using AutoDock Vina. The unit of the scores is absolute value of kcal/mol.

| **ABL1** | **Wild** | **T315I** |
| --- | --- | --- |
| Imatinib | 12.4 | 7.2 |
| Bosutinib | 8.7 | 6.2 |
| Dasatinib | 9.7 | 7.3 |
| Nilotinib | 13.4 | 9.2 |
| Ponatinib | 11.9 | 10.5 |
| Axitinib | 8.9 | 7.3 |
| **EGFR** | **Wild** | **T790M** |
| Erlotinib | 9.5 | 6.7 |
| Gefitinib | 8.9 | 7.7 |

**Supplementary Table S8.** Comparison of docking scores and kinase activity dada in ABL1 and EGFR.

| **ABL1** | **Wild**  **(fitness score)** | **Wild**  **(kd)** | **T315I**  **(fitness score)** | **T315I**  **(kd)** |
| --- | --- | --- | --- | --- |
| Imatinib | 112.65 | 1.1 | 46.35 | NULL |
| Dasatinib | 66.76 | 0.029 | 35.92 | 890 |
| Nilotinib | 119.38 | 10 | 63.39 | 660 |
| Bosutinib | 58.65 | 0.12 | 40.84 | 21 |
| Axitinib | 72.49 | 84 | 68.58 | 3.6 |
| **EGFR** | **Wild**  **(fitness score)** | **Wild**  **(kd)** | **T790M**  **(fitness score)** | **T790M**  **(kd)** |
| Erlotinib | 74.46 | 0.67 | 59.78 | 140 |
| Gefitinib | 79.53 | 1 | 51.15 | 40 |
| **KIT** | **Wild**  **(fitness score)** | **Wild**  **(kd)** | **D816H**  **(fitness score)** | **D816H**  **(kd)** |
| Axitinib | 97.44 | 3.2 | 92.51 | 4200 |
| Linifanib | 70.64 | 2 | 70.19 | 1100 |
| Masitinib | 102.38 | 8.1 | 108.3 | 1500 |
| **RET** | **Wild**  **(fitness score)** | **Wild**  **(kd)** | **V804M**  **(fitness score)** | **V804M**  **(kd)** |
| Cediranib | 75.84 | 6.1 | 61.54 | 3800 |

**Supplementary Table S9**. Pairwise similarity values calculated using MACCS. The scores are the Tanimoto coefficient between two drugs. The closer the Tanimoto coefficient value is to 1, the more similar the structures of the two drugs are.

|  | **Axitinib** | **Imatinib** | **Bosutinib** | **Dasatinib** | **Erlotinib** | **Gefitinib** | **Nilotinib** | **Ponatinib** |
| --- | --- | --- | --- | --- | --- | --- | --- | --- |
| **Axitinib** | 1 | 0.373134 | 0.2875 | 0.358974 | 0.338462 | 0.293333 | 0.481481 | 0.457143 |
| **Imatinib** | 0.373134 | 1 | 0.608108 | 0.671233 | 0.450704 | 0.486842 | 0.6 | 0.761194 |
| **Bosutinib** | 0.2875 | 0.608108 | 1 | 0.621951 | 0.652174 | 0.771429 | 0.38961 | 0.6125 |
| **Dasatinib** | 0.358974 | 0.671233 | 0.621951 | 1 | 0.426829 | 0.5875 | 0.549296 | 0.670886 |
| **Erlotinib** | 0.338462 | 0.450704 | 0.652174 | 0.426829 | 1 | 0.6875 | 0.338235 | 0.467532 |
| **Gefitinib** | 0.293333 | 0.486842 | 0.771429 | 0.5875 | 0.6875 | 1 | 0.402778 | 0.5375 |
| **Nilotinib** | 0.481481 | 0.6 | 0.38961 | 0.549296 | 0.338235 | 0.402778 | 1 | 0.606061 |
| **Ponatinib** | 0.457143 | 0.761194 | 0.6125 | 0.670886 | 0.467532 | 0.5375 | 0.606061 | 1 |

(b)

(a)


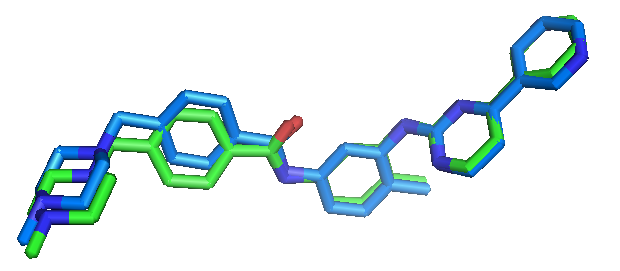

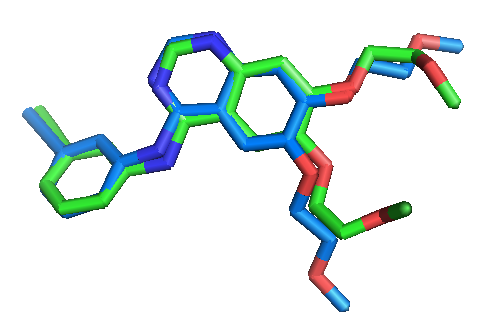
**Supplementary Figure S1**. Re-docking of imatinib and erlotinib in ABL1 and EGFR.(a)Re-docked mode of imatinib(blue) superimposed with the co-crystal(green) in 2HYY(rmsd=0.691). (b)Re-docked mode of erlotinib (blue) superimposed with the co-crystal (green) in 4HJO (rmsd=1.252).

(a)


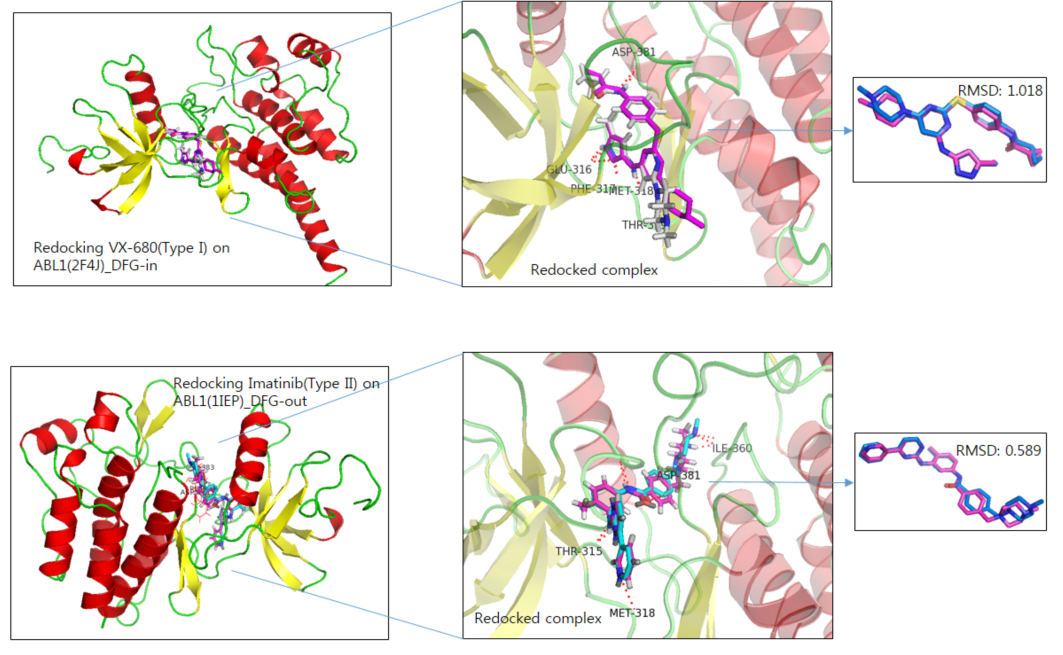


(b)

**Supplementary Figure S2**. The results of re-docked ligands on different DFG states. (a) The actual (blue) and the predicted (magenta) pose of VX-680 in DFG-in state of ABL1 (rmsd=1.018). (b) The actual (blue) and the predicted (magenta) pose of Imatinib in DFG-out state of ABL1 (rmsd=0.589).

1. Martin MP, Alam R, Betzi S, Ingles DJ, Zhu JY, Schonbrunn E. A Novel Approach to the Discovery of Small-Molecule Ligands of CDK2. Chembiochem : a European journal of chemical biology. 2012;13(14):2128-36.
